# Video-based augmented reality combining CT-scan and instrument position data to microscope view in middle ear surgery

**DOI:** 10.1038/s41598-020-63839-2

**Published:** 2020-04-21

**Authors:** Raabid Hussain, Alain Lalande, Roberto Marroquin, Caroline Guigou, Alexis Bozorg Grayeli

**Affiliations:** 1 ImViA Laboratory, University of Burgundy Franche Comte, Dijon, France; 2 Medical Imaging Department, Dijon University Hospital, Dijon, France; 3 Otolaryngology Department, Dijon University Hospital, Dijon, France

**Keywords:** Microendoscopy, Preclinical research

## Abstract

The aim of the study was to develop and assess the performance of a video-based augmented reality system, combining preoperative computed tomography (CT) and real-time microscopic video, as the first crucial step to keyhole middle ear procedures through a tympanic membrane puncture. Six different artificial human temporal bones were included in this prospective study. Six stainless steel fiducial markers were glued on the periphery of the eardrum, and a high-resolution CT-scan of the temporal bone was obtained. Virtual endoscopy of the middle ear based on this CT-scan was conducted on Osirix software. Virtual endoscopy image was registered to the microscope-based video of the intact tympanic membrane based on fiducial markers and a homography transformation was applied during microscope movements. These movements were tracked using Speeded-Up Robust Features (SURF) method. Simultaneously, a micro-surgical instrument was identified and tracked using a Kalman filter. The 3D position of the instrument was extracted by solving a three-point perspective framework. For evaluation, the instrument was introduced through the tympanic membrane and ink droplets were injected on three middle ear structures. An average initial registration accuracy of 0.21 ± 0.10 mm (n = 3) was achieved with a slow propagation error during tracking (0.04 ± 0.07 mm). The estimated surgical instrument tip position error was 0.33 ± 0.22 mm. The target structures’ localization accuracy was 0.52 ± 0.15 mm. The submillimetric accuracy of our system without tracker is compatible with ear surgery.

## Introduction

Middle ear surgery involves manipulation of small, delicate and complex structures inside a very confined workspace. Critical nerves and blood vessels are in close proximity of these structures making submillimetric accuracy, a prerequisite for surgical procedures to be carried out safely^1^. Conventional image guidance systems used in ear surgery have a limited use due to their insufficient precision and poor ergonomics^1^.

In conventional surgery, middle ear contents are approached through external auditory canal after tympanomeatal flap elevation. This approach entails bleeding into the middle ear, risk of tympanic membrane perforation or lateralisation, injury to ossicles or corda tympani and postoperative care for several days^2^. Alternatively, transtympanic procedures have been designed to access middle ear cleft structures through a small puncture in the tympanic membrane which would spontaneously heal as during a grommet insertion. This route has been employed for different indications such as ossicular chain repair, drug administration and labyrinthine fistula diagnosis^3–5^. The procedure offers several potential advantages over traditional surgery: faster route, tympanic membrane preservation, reduced bleeding, simpler and less painful post-operative care. However, manipulation of fragile ossicles through this keyhole approach will probably require a robot-based technique^6,7^. Moreover, visualization of middle ear content and surgical instruments behind the closed tympanic membrane is essential. This goal may be achieved through combination of middle ear CT-scan and real-time microscopic video in an augmented reality (AR) framework.

Different works have been proposed on AR based surgical systems mainly targeting orthopedics, hepatobiliary and neurologic surgeries^8^. However, only few studies have targeted cranial base and otolaryngology domains owing to high precision requirements and intricate anatomy^9,10^. Particularly in ear surgery, Lee *et al*. projected real-time middle and inner ear OCT images onto the microscope view^11^. However, owing to the characteristics of OCT imaging, the working distance from the objected lens to the surgical site needs to be sufficiently maintained. Wisotzky *et al*. proposed an AR system visualizing depth information in the ear cavity using a color coded scheme^12^. Both of the above systems mainly augment depth information about different structures. Liu *et al*. performed robotic cochleostomy using DaVinci surgical system with visualization of critical structures using an AR system^13^. However, DaVinci surgical system does not come with appropriate tools for ear surgery. Alternatively, a dedicated otologic robotic system such as Robotol (Collin Medical SAS, France) might be useful^14^. Moreover, in most studies the set-up required a conventional tracking system (mechanical, optical or electromagnetic). Similarly, in cranial base domain, mutual information, contour-based and point-based registration methods such as ICP have been widely used^10,13,15–18^. Clinically, 5–10 minutes registration time has been regarded as acceptable with submillimetric precision^19^. Moreover, anatomical landmarks are difficult to ascertain and track once the procedure starts as they may shift or become obscured from fluids or instruments. Different systems have adopted external tracking systems like optical or electromagnetic trackers to track the motion between the patient and the camera^10,16,20,21^. However, they are expensive and bulky. Alternatively, image-based algorithms exploiting optical flow and image features are being developed and incorporated into the AR setup^19,22–24^. When optimized, these methods appear to be more ergonomic to apply with a simpler system setup. In this study, we aimed at evaluating the potential application of these methods to the AR in otological surgery.

Additionally, information related to surgical instrument pose behind closed tympanic membrane must be provided to the surgeon. The issue of instrument visualization in laparoscopic surgery has been already investigated. Different techniques have been developed to identify the instruments in the microscope frame based on pre-known kinematic information, instrument templates, visual cue models and artificial markers^25,26^. However, limited work has been reported on the tri-dimensional pose estimation (position and orientation) of instruments. Some examples are the use of random forest classifiers with instrument geometry as a prior, vision-based robot control techniques, fiducial marker points, and 3-collinear perspective frameworks^27–29^. In ear surgery, the small size of the target structures requires submillimetric precision^30^. As a proof of concept, it has been shown that AR combining otoendoscopy video and CT-scan may provide this level of precision in the middle ear if a careful registration is manually conducted by an expert^22^.

In a previous work, the applicability and performance of different tracking processes (using both endoscope and surgical microscope) was assessed on both human cadaveric temporal bones and artificial temporal bones^15,22^. The aim of this study was to develop and assess a real-time AR system combining CT-scan data and microscopic video of the ear canal together with visualization of the surgical instrument behind closed tympanic membrane. This article extends our previous studies on AR based transtympanic procedures^15,22^ by (1) validating the system’s tracking schemes in near-realistic and challenging scenarios, and (2) imitating an actual procedure (drug administration), and (3) assessing the work in real-time instead of employing a recorded video. These developments brought the system several steps closer to its application in the operating room. To our knowledge, no other work has been reported on AR-based transtympanic procedures.

## Material and methods

### Experimental setup

Six artificial human temporal bone specimens (Phacon Inc., Leipzig, Germany), with variations in corresponding age, size and anatomy, were included in this prospective study. Ethical approval and informed consent was not required for this study. Five or six fiducial markers (0.5 mm diameter and 1 mm long stainless-steel wire) were glued to the periphery of the tympanic membrane, evenly distributed on its perimeter (Table 1). To optimize image to object registration, the markers were placed far apart in a non-linear configuration with their combined centre coinciding with the projection of the target on the plane defined by the markers^31^.

**Table 1:** Experimental conditions.

Experiment Number	1	2	3	4	5	6
Phantom model	TF-ba	TF-ka	TF-bm	TF-ec	TF-dc	TF-bm
Corresponding patient age	55	1	55	6	2	55
Number of markers used	5	6	5	5	6	5
X, Y translations	Yes	Yes	Yes	Yes	Yes	Yes
Z translations	Yes	Yes	Yes	No	Yes	Yes
Rotation	Yes	Yes	Yes	Yes	Yes	No
Movement speed	Low	High	High	High	Low	High

The experiments were performed for a duration of two minutes each, using fiducial markers as reference points for evaluation. During experiments, estimated slow (<5 mm/s) or rapid (5–10 mm/s) translations, rotations and pitches were applied to the microscope. Please refer to the Phacon Inc. website for further details about the temporal bone phantoms (https://www.phacon.de/en/hno/felsenbein).

**Figure 1:**
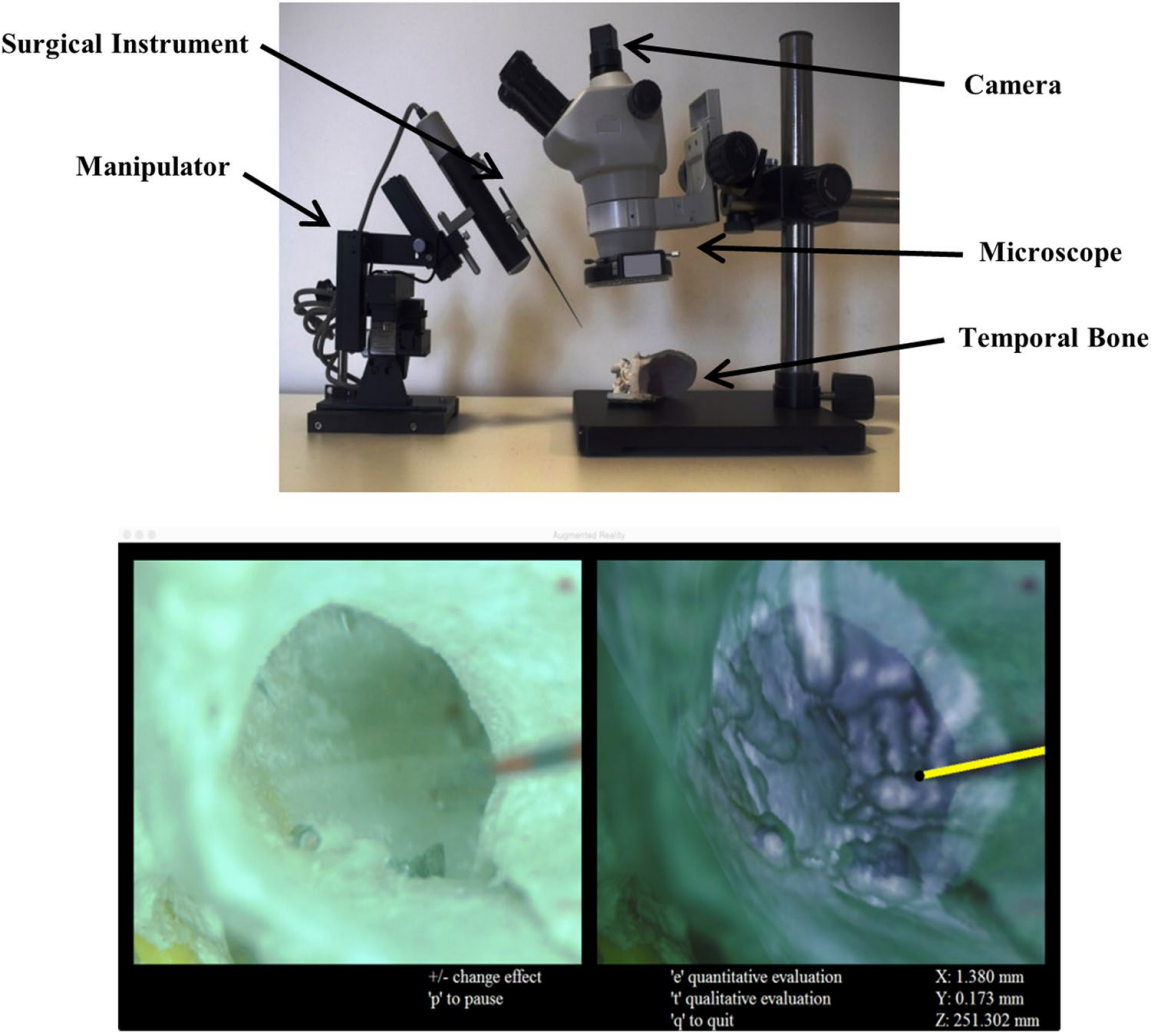
Experimental setup. A surgical microscope connected to a digital camera was placed over the temporal bone (top panel). The micromanipulator was attached to a micro instrument and simulated the keyhole surgery. On the computer screen (below), the real-time video from the microscope (lower left panel) and the augmented reality window (lower right panel) can be observed. Two of the marker points on the instrument are visible on the real-time video. The instrument is displayed in yellow on the augmented reality window and the 3D pose of the instrument is provided in mm on the bottom right corner of the display.

All specimens underwent pre-operative CT-scan (0.6 × 0.6 × 0.3 mm^3^ voxel size, General Electric Medical Systems, Buc, France). 3D reconstruction, based on DICOM data, of middle ear cleft was carried out using Osirix virtual endoscopy function (Pixmeo SARL, Bernex, Switzerland). The 3D reconstruction was obtained by placing the virtual endoscope in the external auditory canal facing the umbo 10 mm outside the tympanic membrane. This image of the middle ear cleft structures was used as reference to warp around the microscope video. In parallel, otoendoscopy was performed for all temporal bone specimens with a surgical microscope (Zoom Pro 10.76, 115 mm working distance, 8–50x zoom, Perfex, Escalquens, France) connected to a high definition camera (xiQ MQ013CG-ON, Ximea Gmbh, Munster, Germany) to visualize the tympanic membrane (Fig. 1). A surgical microneedle was introduced into the middle ear through a puncture hole in the tympanic membrane. It was controlled by a micromanipulator (DC3314R, World Precision Instruments, Sarasota, FL, USA) with 3 degrees of freedom and a 37 × 20 × 20 mm^3^ workspace with 0.1 mm precision.

**Figure 2:**
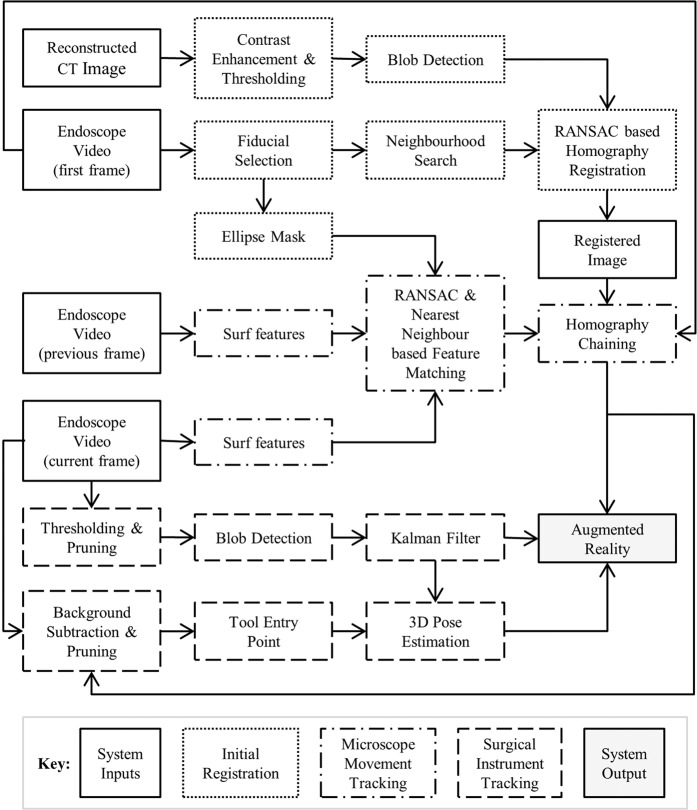
Flow diagram of the proposed methodology. Sub-processes belonging to each main step are grouped together with similar box styles. See text for details.

AR was implemented by combining real-time video images of the external auditory canal and the tympanic membrane to the 3D CT-scan reconstruction of the middle ear cavity (Fig. 1). The software was developed using OpenCV, Eigen libraries and Ximea API in XCode (C + + ). The program was run on an iMac computer (2.9 GHz Intel Core i5 processor, 8 GB 1600 MHz DDR3 RAM, NVIDIA GeForce GT 750 M 1024MB graphic card, OSX Yosemite 10.10.5 operating system). The system involved 3 main processes: initial registration, microscope movement tracking and instrument identification (Fig. 2).

The inputs of the system were the following:

1. The video was the real-time film of the tympanic membrane acquired through the microscope (Fig. 3a).

**Figure 3:**
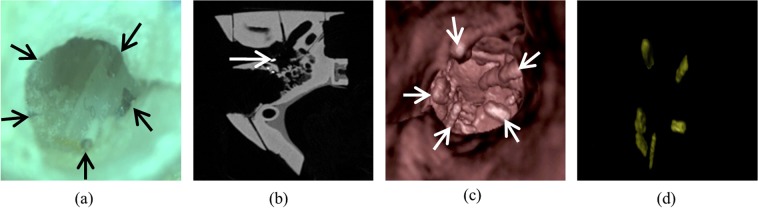
Augmented reality system inputs. System inputs with five attached fiducial markers (indicated by arrows) that appear (**a**) grey on the microscopic image, (**b**) white on the CT-scan axial view, and (**c**) as protrusions on the virtual endoscopy image based on CT-scan. (**d**) Automatic extraction of markers from the virtual endoscopy image.
2. The camera matrix represented extrinsic and intrinsic properties of the camera. The focal length of the camera, used in 3D pose estimation, was determined using Zhang’s algorithm^32^.
3. The reconstructed middle ear image (behind the tympanic membrane) was obtained from the preoperative CT scan through Osirix’s 3D virtual endoscopy function (Fig. 3c).

### Initial registration

**Figure 4:**
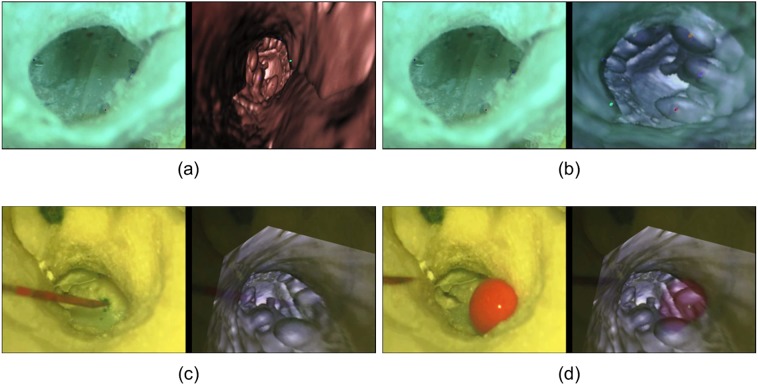
Different processes of the augmented reality system. (**a**) Registration point selection. (**b**) Virtual image warped over microscope video after registration. (**c**) AR system before fluid injection. (**d**) AR system after fluid injection.

This first step consisted of registering the reconstructed CT-scan image to the real-time microscopic image of the tympanic membrane extracted from the video. From the CT-scan image, fiducial markers were extracted using contrast enhancement and thresholding (Fig. 3(d)). Marker centre points, obtained by detecting blob-like contours in the image using topological structural analysis^33^, were highlighted on the reconstructed image for assistance (Fig. 4(a)). Their corresponding points were manually selected in the microscopic image. The reconstructed image was then warped onto the microscopic image using RANdom SAmple Consensus (RANSAC) based homography^34,35^:

1 $${P}_{i}\approx {H}_{R}{P}_{i}^{{\prime} }$$

where *H*_*R*_ is the registration matrix, *P*_*i*_’ are the detected marker points in CT image and *P*_*i*_ are their corresponding points in microscope image determined using a RANSAC approach. This algorithm finds the best possible correspondence between points in a small neighbouring window of the selected fiducial points to minimize the registration error. From Eq. (1), *H*_*R*_ can be determined by minimizing the error function. An ellipse shaped mask (used to filter out non planar features in microscope tracking) was also extracted using these corresponding marker points.

A blend operator was also integrated into the system to allow the user to control the opacity of the registered CT image over the microscope video during surgery (as per requirements):

2 $${I}_{AR}=\beta {I}_{M}+(1-\beta ){I}_{CT}$$

where *I*_*AR*_ is the augmented reality output, *I*_*M*_ is the microscope image, *I*_*CT*_ is the registered CT image and *β ∈ [0,1]* is the blend factor.

### Microscope movement tracking

A robust estimation of the operative microscope movements solely based on image features was developed in order to maintain correspondence with the CT-scan image^22^. A tracking scheme, comprising of RANSAC and nearest neighbour based Speeded-Up Robust Features (SURF) matching process was employed to determine transformation between consecutive frames^36–38^. SURF is an algorithm which uses different mathematical formulations to extract information about key points in an image (e.g. corners and edges). The feature-matching algorithm compared all the key points between consecutive frames using random sampling based on feature distance. Any key point that had more than one close matches was not considered for determining the transformation. The ellipse-shaped mask, generated in the initial registration step using fiducial marker centre points, was used to further refine the transformation by filtering out any non-planar features present outside the eardrum^22^. A chained homography framework (cumulating the transformations between all the previous frames:

3 $$H={H}_{T}H$$

where *H* is the cumulative homography and *H*_*T*_ is the transformation between current and previous frames) was used to warp the registered reconstructed image onto the current microscopic frame.

### Surgical instrument tracking

Three collinear colour markers were painted on the surgical instrument. The proposed instrument identification approach assumed that no instrument was present in the first microscope frame. The first frame underwent a transformation based on the homography *H* and was subtracted from the current frame to obtain an approximation of the area occupied by the instrument. A pruning step was carried out to eliminate any false positive regions (due to discrepancies in *H*). If only a small area was obtained (less than a threshold), it indicated that no instrument was present in the current frame. Otherwise, the instrument entry point was then searched in the approximated instrument region (on the frame boundary points only). The collinear markers were extracted from the image using colour thresholding followed by pruning. However, due to small focus range of the microscope, the extraction was not perfect, and the centre points were extracted using blob detection^15^. The tool entry point was then used to associate the marker centres to marker labels *B*, *C* and *D* where *B* is closest to the instrument tip *A*, and *D* is closest to the tool entry point. A Kalman filter was used to refine the marker centre points, eliminating any residual degradation caused by the blurring effect. This filter is a mathematical algorithm which estimates the state of a system from its dynamic model and a series of partial or distorted observed measurements over time^38^. The instrument tip location can then be deduced as:

4 $$a=\frac{1}{3}\left(b+c+d+\frac{AB}{CD}(c-d)+\frac{AC}{BD}(b-d)+\frac{AD}{BC}(b-c)\right)$$

where *a* is the projection of the instrument tip *A* on the 2D image frame, *b*, *c* and *d* are projections of the markers and alphabet pairs represent physical distances between corresponding markers. Three-point perspective framework^39^ was used to estimate 3D pose of the instrument. By setting the focal length of the camera as the z coordinate of the image projection points (b, c and d) and measuring the physical distance between markers (AB, BC, CD), the position of the instrument tip could be estimated using Eq. (4), by fitting the physical geometry (3D) of the tool onto the projected lines *Ob*, *Oc* and *Od*, where *O* is the origin of the camera axis^15^.

### Evaluation

A quantitative analysis was performed to evaluate the registration process which involved computing the distance between the positions of fiducial points input by the user and their corresponding points (after registration). The root mean square error was used to quantify the results.

The tracking accuracy of the system was evaluated every 30 seconds for 2 minutes. The microscopic view was translated, rotated, zoomed-in and out with an approximate speed of 5–10 mm/s during this period (Table 1). The distances between the “real” positions of the markers and their estimated positions (by the system) were checked. The word “real” was used because these points were computed using template matching algorithm. This algorithm consisted of taking the neighbourhood of the corresponding point as a template and estimating its location in the current frame based on different transformations (scaling, translation and rotation).

For evaluation of the surgical instrument tracking, the position provided by the robotic manipulator attached to the instrument was used as the reference: Pre-known displacements of 2, 4 and 6 mm were applied, independently in each optical axis, using the micromanipulator. Their corresponding displacements detected by the system were measured and the instrument tracking error was computed as the root mean square error of the difference. Averages of 50 samples per individual displacement were recorded for analysis. Additionally, a statistical one-way ANOVA test was carried out to compare the inter-axis pose estimation performance in each individual axis. A p-value <0.05 was considered as significant.

A second series of experiments were performed to further assess the performance of the system. In these five experiments, the movement tracking was assessed for a longer time period (8 minutes) with different experimental conditions to further approach surgical conditions. A surgical microscope (Zeiss OPMI MDO S5 Microscope, Ziess, US) was employed for these experiments. The experiments were performed in different lighting conditions and liquid red ink was introduced to simulate hemorrhage. Movements similar to previous set of experiments were applied to the microscope and the tracking accuracy was measured accordingly. Experimental conditions for these experiments are listed in Table 2. One-way ANOVA test was carried out to compare the tracking results between different experiments. A p-value <0.05 was considered as significant.

**Table 2:** Additional experimental conditions.

Experiment Number	A1	A2	A3	A4	A5
Phantom model	TF-ba	TF-dc	TF-bm	TF-bm	TF-bm
Corresponding patient age	55	2	55	55	55
Number of markers used	5	5	5	5	5
Movement speed	High	High	Low	Low	High
Ambient lighting	Medium	Low	Low	Medium	Medium
Microscope light	High	Medium	Medium	High	High
Comments	1. External object (wire) was introduced between 3 and 4 minutes2. Big jerk was applied at 2:15 minutes requiring re-registration	—	—	1. External object (needle) was introduced between 3 and 6 minutes2. Liquid ink was at introduced at 5 minutes	Liquid ink was present throughout the experiment

Additional experimental conditions. The experiments were performed for a duration of eight minutes each, using fiducial markers as reference points for evaluation. During experiments, translations, rotations and pitches were applied to the microscope. Further details on temporal bone phantoms are available at https://www.phacon.de/en/hno/felsenbein.

In three additional experiments (phantom models: TF-bm, TF-ba, TF-dc), the instrument was firstly placed on the umbo and then introduced into the middle ear space through a small puncture in the tympanic membrane. The tip was placed on the extremity of the long process of incus and finally the highest point on the round window niche. Micro-droplets of ink were injected at these target points. The tympanic membrane was then removed and locations of the droplets were verified by computing the distance between their actual locations and the expected ones.

## Results

The system remained stable in all cases throughout the experiments (see Supplementary Video S1). Different stages of the experimental study are depicted in Fig. 4 and the augmented reality display window is depicted in Fig. 1. A global mean image refresh rate of 12 ± 1 frames per second (fps) was obtained.

Fiducial marker based initial registration eased and speeded up the process of corresponding point selection. A mean registration error of 0.21 ± 0.10 mm (n=6) with a mean registration time of 5.57 ± 2.65 seconds (range: 1.2–8.3 seconds) was noted.

**Figure 5:**
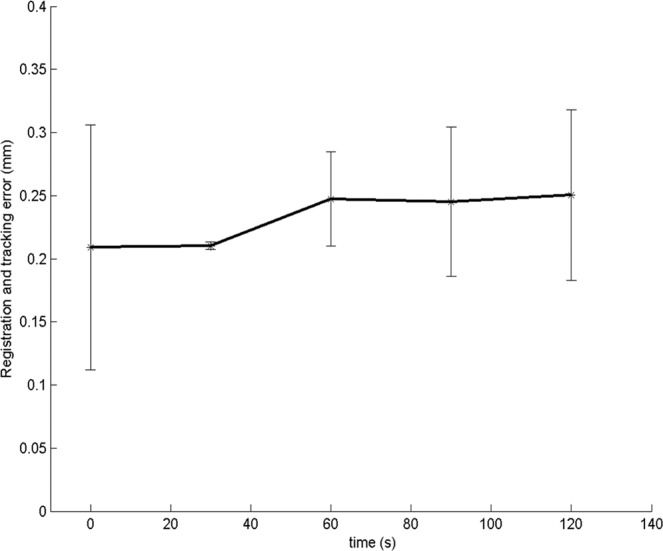
Mean initial registration and tracking errors during the 2 minute tracking of microscope movements. Values represent mean ± standard deviation (n = 6).

**Figure 6:**
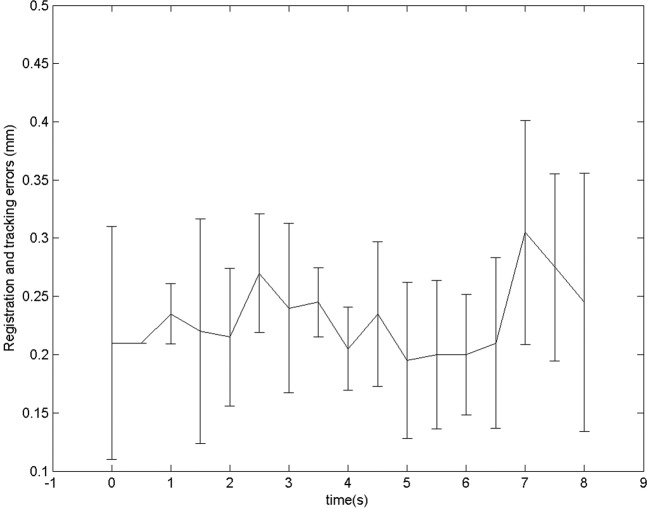
Tracking errors for additional experiments during the 8 minute tracking in different experimental conditions using a surgical microscope.

The microscope tracking process also yielded a sub-millimetric drift (0.04 ± 0.07 mm at 120 seconds), suggesting a very slow propagation error (Fig. 5). Similarly, in additional experiments (with simulated surgical conditions), an average drift of 0.04 ± 0.11 mm at 8 minutes was obtained (Fig. 6). The system maintained synchronization in all the experiments. No significant difference in the performance was observed between experiments (p-value, non-significant). During experiment A1, a sudden jerk was applied at 2.15 minutes to the microscope in order to check the limitations of the system. Consequently, a re-registration was required as the system could not comprehend extreme movements (such as jerks). In experiment A4, liquid ink covered two of the registration points which were used for determining the registration and tracking errors. Thus, the performance could only be evaluated qualitatively. The experiment with the introduction of liquid red ink can be seen in Fig. 4(c,d) and Supplementary Video S1.

The surgical instrument was also accurately identified. In different experiments, a small oscillatory instrument movement was observed. Different displacements were applied in each individual axis and the 3D pose was estimated (Table 3). A mean instrument tip position error of 0.19 ± 0.05 mm (n = 150) in X-axis, 0.19 ± 0.02 mm (n = 150) in Y-axis and 0.55 ± 0.46 mm (n = 150) in Z-axis was observed. Tukey HSD post-hoc test revealed that the pose estimation in X and Y axes was acceptable and significantly better than the estimation in Z axis (*p-value* < *0.05* when compared with both X and Y axes) as small deviation in instrument identification constitutes a large deviation in Z axis pose estimation. No significant difference was observed between X and Y axes pose estimations. The mean pose estimation error $(\sqrt{{X}^{2}+{Y}^{2}+{Z}^{2}})$ was 0.33 ± 0.22 mm (n = 450).

**Table 3:** Accuracy of surgical instrument tracking.

Movement Direction	Actual Displacement		
	2 mm	4 mm	6 mm
**X (mm)**	2.13 ± 0.01	3.75 ± 0.04	6.18 ± 0.10
**Y (mm)**	1.95 ± 0.04	4.07 ± 0.01	5.54 ± 0.02
**Z (mm)**	2.35 ± 0.56	3.65 ± 0.21	6.96 ± 0.61

3D pose estimation of the microneedle after predetermined displacements by a micromanipulator. Data represent mean ± standard deviation of 50 samples for each direction and displacement.

**Figure 7:**
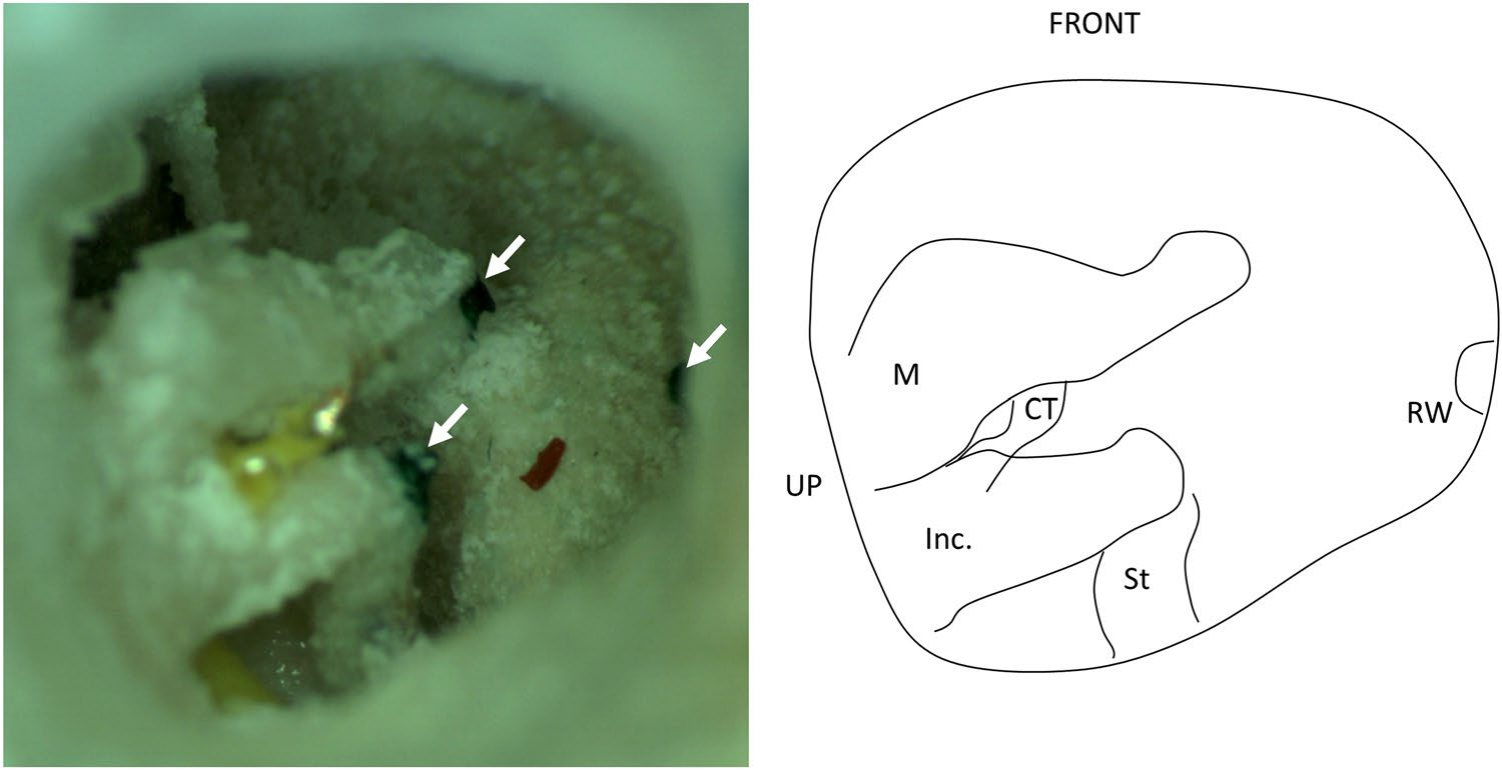
Qualitative analysis of the localisation of the injected droplets. A right temporal bone (TF-ba) is shown in operative position. The black marker dots (arrow heads) represent the targets where the ink droplets were injected. CT: Corda tympani, Inc.: Incus, M: malleus, RW: round window, St: Stapes.

Similarly, the target structures were accurately reached with mean localization errors (n = 3) of 0.56 ± 0.14 mm, 0.54 ± 0.16 mm and 0.46 ± 0.19 mm for umbo, incus tip and round window niche, respectively (Fig. 7). The mean target error was 0.52 ± 0.15 mm (n = 9).

## Discussion

In this study, we showed that a marker-based AR system combining preoperative CT image with real-time 2D video from the operative microscope, based only on computer vision and without any tracking system techniques, is possible. A 3D reconstructed view of the CT-scan was registered to the microscopic view based on homography transformation. The system employed algorithms from different domains (e.g. image processing, visual perception, endoscopy, radiology and autonomous navigation). It provided additional visual information on the middle ear structures and the surgical instrument with submillimetric precision, compatible for middle ear surgery.

In previous works, the performance of the motion tracking with manual initial registration was analysed on phantom and cadaver subjects^15,22^. Similar results were obtained for both types of subjects. This study, provides crucial steps toward the applicability of AR on middle ear in the operating room by enhancing the registration step, enabling the system to process the video in real-time and to automatically detect instruments. The CT to video registration appears to be crucial, since errors during this step will propagate throughout the process. Indeed, in most computer-assisted surgical systems, image registration plays paramount role in the overall performance of the system. In endoscope-CT registration, combinations of different intensity-based schemes such as cross-correlation, squared intensity difference, pattern intensity, normalised and gradient mutual information have shown promising results^40,41^. Similarly, feature-based schemes involving natural landmarks, contour based feature points, iterative closest point and k-means clustering have also been exploited^42–44^. The main challenge of our system was the low similarity between the multi-modal images. To overcome this, artificial markers that increase visibility and lower the perturbation were introduced^45^. Indeed, very few natural landmarks are visible on both CT and during otoscopy around the tympanic membrane, making introduction of markers beneficial in terms of both registration time (10–15 seconds vs 55–80 seconds) and accuracy (0.21 mm vs 0.25 mm) as compared to manual registration^22^. In the current setup, the markers were placed arbitrarily around the periphery of the tympanic membrane which was assumed to represent a near-planar surface. As a future step for clinical trials, patient specific custom rings in contact with the tympanic membrane can be designed to house the markers in an ergonomic and robust manner. Even with millimetric markers, finding the exact corresponding points is practically infeasible. This limitation was overcome by implementing a RANSAC algorithm in the registration process. This mathematical algorithm allows estimating the parameters of a model with iterative measures and possible aberrant values. In our model, the algorithm took into account similar points in a small neighbouring window around the selected fiducial markers and provided the best matching solution^35^.

Another challenge was to maintain correspondence between CT and video by tracking the microscope movements. In routine applications, the microscope will be quasi-static. However, in order to validate the robustness of the system, various movements were applied to the microscope. With the use of SURF algorithm, a minimal propagation error was observed during tracking, even when intricate motion was applied, allowing lengthy surgical procedures. Since the tracking was based on image features, the fiducial markers do not need to be visible in the surgical video after registration step has been successfully carried out and this represents a potential advantage in terms of ergonomics. Virtual objects introduce additional unwanted occlusions leading to loss of internal organ information in an AR system. The blend operator allows the surgeon to turn off or decrease the opacity of virtual image when it is not required. Moreover, the raw video from the microscope is also available to the surgeon next to the augmented reality display. Furthermore, to address the loss of information on internal organs, a combination of transtympanic endoscopy and AR may be utilized in the operating room, in order to validate the AR information and to explore details that are less visible on CT-scan data such as adhesions. Transtympanic endoscopy has already been evaluated in similar key-hole procedures^4^.

Keyhole surgery cannot be performed without instrument depth information inside the middle ear and behind the tympanic membrane. Under operative microscope, conventional computer vision approaches exploiting natural features like gradient information or greyish nature of the surgical instruments are bound to fail as the perception range of microscopes is limited. In addition, since the instrument may enter from any direction and protrude indefinitely, so geometric priors may not be valid. Our proposed method, using colour markers, took into account such specifications of the otologic surgery. Although the markers can be placed anywhere on the instrument, the segment containing the markers needs to remain in the video frame for accurate pose estimation. However, this method cannot determine instrument pose angle in the optical axis, without introduction of additional priors e.g. coplanar markers.

The accuracy of the system on 3 middle ear target structures was submillimetric and this level of precision is essential for otologic procedures. This is the most important performance factor as it encompasses all different aspects of the system: precision of CT reconstruction, registration, motion and instrument tracking. This performance may be improved by integrating additional 3D information about target structures.

## Conclusion

In conclusion, the proposed AR system based only on computer vision techniques provided a precise vision of the middle ear contents and the surgical instrument behind the closed tympanic membrane in real-time with a high image fresh rate. The system maintained correspondence between CT-scan and video during microscope movements. This technique opens insights to different transtympanic procedures such as drug administration, labyrinthine fistula repair and ossicular chain reconstruction by a transtympanic keyhole approach.

## Supplementary information

Supplemental Video S1.

Supplementary information.

## Data Availability

The data i.e. the phantoms that support the findings of this study are available from Phacon Inc., Leipzig, Germany (https://www.phacon.de/en/hno/felsenbein).
