# Supplementary material for: Video-based augmented reality combining CT-scan and instrument position data to microscope view in middle ear surgery
Source: Sci Rep. 2020 Apr 21;10:6767. doi: 10.1038/s41598-020-63839-2 (PMC7174368; doi:10.1038/s41598-020-63839-2)
Supplement: Supplementary file 2 — Supplementary information. [file 41598_2020_63839_MOESM2_ESM.pdf]

Video-based augmented reality combining CT-scan and instrument position data to microscope view in middle ear surgery

Raabid Hussain, Alain Lalande, Roberto Marroquin, Caroline Guigou, Alexis Bozorg-Grayeli

Supplementary Information File

List of Supplemental Content

Supplemental Video S1: A video that demonstrates different processes of the proposed system.
